# The Riffomonas Reproducible Research Tutorial Series

**DOI:** 10.21105/jose.00013

**Published:** 2018-08-30

**Authors:** Patrick D Schloss

**Affiliations:** 1 Department of Microbiology & Immunology, University of Michigan, Ann Arbor, MI USA

## Summary

The Riffomonas Reproducible Research tutorial series is a collection of tutorials that focuses on the improvement of reproducible data analysis for those doing microbial ecology research. Although the materials focus on issues in microbial ecology, the principles are broadly applicable. Each tutorial presents broad concepts and how they are related to reproducibility as well as applied practice using specific tools that are designed to foster reproducibility. In addition to activities that are specific to individual tutorials, the series also uses data from Kozich et al. (Kozich, Westcott, Baxter, Highlander, & Schloss, 2013) to create an example that is developed over the course of fourteen tutorials. The tutorial series consists of 14 lessons that are available as HTML-based presentations with embedded presenter notes taken from live demonstrations of the content that are available as videos hosted on YouTube.

## Statement of Need

The design and structure of the tutorials in the series give scientists new to the field of microbial ecology research the background and tools that they need to implement a reproducible analysis of their data. Although there are a growing number of commentaries speak to a “reproducibility crisis” in science and microbiology in particular (Casadevall, Ellis, Davies, McFall-Ngai, & Fang, 2016; Collins & Tabak, 2014; Garijo et al., 2013; Noble, 2009; Ravel & Wommack, 2014; Schloss, 2018), there has not been a concentrated effort to improve reproducibility within microbiology. Although the results of these studies are exciting, the interdisciplinary nature of the field means that the practitioners are not experts in all areas. This is especially true in the analysis of the large datasets that are being generated using high throughput sequencing.

## Content

The fourteen tutorials focus on issues related to documentation, transparency, openness, and automation. The primer by Noble (Noble, 2009) motivates much of the material on project organization, documentation, and automation. Because of the unique nature of computational research relative to bench science, considerable attention is also given to using version control to document the evolution of a project and how to best work individually as well as with others to foster reproducibility. Although the themes are generally tool-agnostic, the practical implementation of these tools is prominent in the materials and when specific tools are used, other options are mentioned. Specific tools that are included in the tutorials include:

- Documentation (markdown, rmarkdown (Allaire et al., 2018), R (R Core Team, 2018), make, git)
- Organization (bash, HPC/AWS, git)
- Automation (bash, R (R Core Team, 2018), make)
- Transparency (ORCID, FigShare, git, GitHub, open source licensing)
- Collaboration (git, GitHub, open source licensing)

The focus of this tutorial series is on developing proficiency with a set of tools to foster reproducible research. Therefore although these tools have many uses, the materials focus on those features of the tools that support greater reproducibility. Specifically, the following tools are used in the tutorials, but learners should not finish the materials expecting to be proficient in using them. Although proficiency in these tools is not required, background in these tools will be helpful.

- R (R Core Team, 2018)
- mothur (Kozich et al., 2013)

Throughout the tutorial there are exercises that encourage the participants to discuss topics with their mentors and research group, activities where the participants are asked to engage resources at a deeper level. By demonstrating a meaningful level of participation in the tutorial series, participants receive a “virtual badge” and will be listed on the Reproducible Research Tutorial Series Honor Roll, which provides a certification of their training. The material is presented in an escalating manner such that over multiple tutorials participants are introduced to a topic, then given partial exposure and deeper exposure, and finally work with the tools proficiently.

The tutorial series is available as a series of slide decks written in markdown and are rendered using the remark framework. The content is available under the CC-BY-SA 4.0 license. The slides are available on the tutorial series GitHub repository. Accompanying the slides are YouTube-hosted videos where the author presents the material and does live-coding demonstrations to help participants engage with the material. In addition to links to the videos on the tutorial home page, there is a YouTube playlist that aggregates the 14 tutorials as the Riffomonas Reproducible Research Tutorial Series. Transcripts from these videos are available as presenter notes for each of the slide decks by pressing ‘p’ when the slides are open.
